# Transcriptional Regulation of VEGF-A by the Unfolded Protein Response Pathway

**DOI:** 10.1371/journal.pone.0009575

**Published:** 2010-03-08

**Authors:** Rajarshi Ghosh, Kathryn L. Lipson, Karen E. Sargent, Arthur M. Mercurio, Joan S. Hunt, David Ron, Fumihiko Urano

**Affiliations:** 1 Program in Gene Function and Expression, University of Massachusetts Medical School, Worcester, Massachusetts, United States of America; 2 Program in Molecular Medicine, University of Massachusetts Medical School, Worcester, Massachusetts, United States of America; 3 Department of Cancer Biology, University of Massachusetts Medical School, Worcester, Massachusetts, United States of America; 4 Department of Physical and Biological Sciences, Western New England College, Springfield, Massachusetts, United States of America; 5 Department of Anatomy and Cell Biology, University of Kansas Medical Center, Kansas City, Kansas, United States of America; 6 Skirball Institute, New York University School of Medicine, New York, New York, United States of America; 7 Institute of Metabolic Sciences, University of Cambridge, Cambridge, United Kingdom; Roswell Park Cancer Institute, United States of America

## Abstract

**Background:**

Angiogenesis is crucial to many physiological and pathological processes including development and cancer cell survival. Vascular endothelial growth factor-A (VEGFA) is the predominant mediator of angiogenesis in the VEGF family. During development, adverse environmental conditions like nutrient deprivation, hypoxia and increased protein secretion occur. IRE1α, PERK, and ATF6α, master regulators of the unfolded protein response (UPR), are activated under these conditions and are proposed to have a role in mediating angiogenesis.

**Principal Findings:**

Here we show that IRE1α, PERK, and ATF6α powerfully regulate VEGFA mRNA expression under various stress conditions. In *Ire1α^−/−^* and *Perk^−/−^* mouse embryonic fibroblasts and ATF6α-knockdown HepG2 cells, induction of VEGFA mRNA by endoplasmic reticulum stress is attenuated as compared to control cells. Embryonic lethality of *Ire1α−/−* mice is due to the lack of VEGFA induction in labyrinthine trophoblast cells of the developing placenta. Rescue of IRE1α and PERK in *Ire1α^−/−^* and *Perk^−/−^* cells respectively, prevents VEGFA mRNA attenuation. We further report that the induction of VEGFA by IRE1α, PERK and ATF6 involves activation of transcription factors, spliced-XBP-1, ATF4 and cleaved ATF6 respectively.

**Conclusions/Significance:**

Our results reveal that the IRE1α-XBP-1, PERK-ATF4, and ATF6α pathways constitute novel upstream regulatory pathways of angiogenesis by modulating VEGF transcription. Activation of these pathways helps the rapidly growing cells to obtain sufficient nutrients and growth factors for their survival under the prevailing hostile environmental conditions. These results establish an important role of the UPR in angiogenesis.

## Introduction

Productive folding of secretory proteins in the endoplasmic reticulum (ER) is essential to ensure normal cell function. In order for secretory proteins to fold properly, ER homeostasis must be maintained. ER homeostasis is defined by the dynamic balance between the ER protein load and the ER capacity to process this load. ER homeostasis can be perturbed by pathological processes such as hypoxia, glucose deprivation, viral infections, environmental toxins, inflammatory cytokines, and mutant protein expression, as well as by physiological processes such as aging. Disruption of ER homeostasis causes accumulation of unfolded and misfolded proteins in the ER. This condition is referred to as ER stress. Cells cope with ER stress by activating the Unfolded Protein Response (UPR) [1], [2]. The UPR is initiated by three ER transmembrane proteins: Inositol Requiring 1 (IRE1), PKR-like ER kinase (PERK), and Activating Transcription Factor 6 (ATF6). These three master regulators sense and interpret protein folding conditions in the ER and translate this information across the ER membrane to regulate downstream effectors. These effectors have two distinct outputs, homeostatic and apoptotic. Homeostatic outputs are adaptive responses that function to attenuate ER stress and restore ER homeostasis. These responses include the attenuation of protein translation to reduce ER workload and prevent further accumulation of unfolded proteins, upregulation of molecular chaperones and protein processing enzymes to enhance the ER folding activity, and the increase in ER-associated degradation (ERAD) components to promote clearance of unfolded proteins. When ER stress reaches a point where the cells cannot tolerate the load of unfolded proteins any more, apoptosis sets in [1].

Recent studies have indicated that cells suffering from insufficient blood supplies experience ER stress. The ER needs energy and oxygen for the folding process, thus nutrient deprivation (low ATP production) and hypoxia caused by insufficient blood supply leads to inefficient protein folding and ER stress in cells, especially in cancer cells that grow and spread rapidly [3], [4], [5]. This condition also occurs in the development of the placenta [6], [7], [8]. Both nutrient deprivation and hypoxia stimulate the production of vascular endothelial growth factor (VEGF) and other angiogenic factors, leading to protection against ischaemic injury [9], [10], [11]. Here we report that the three master regulators of the UPR, IRE1α, PERK and ATF6α, mediate transcriptional regulation of VEGFA under ER stress which occurs during normal development of labyrinthine trophoblast cells in the placenta as well as in cancer cells.

## Results

### VEGFA Expression Is Increased by ER Stress

**Figure 1:**
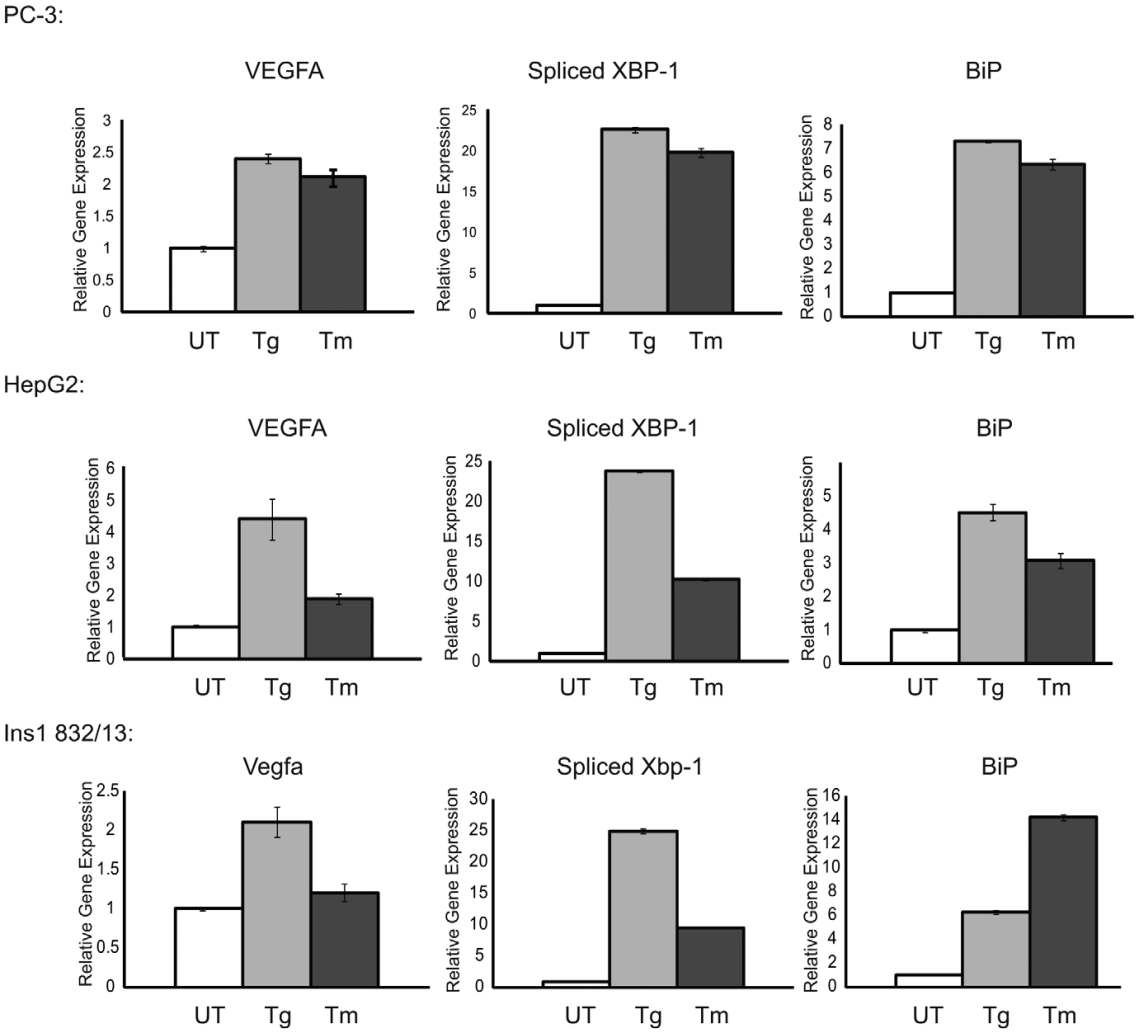
VEGFA mRNA expression is induced by ER stress. Expression levels of VEGFA, Spliced Xbp1 and BiP were measured by quantitative PCR in PC3 cells, HepG2 cells and INS-1 832/13 cells following treatment with thapsigargin (Tg, 1 µM), tunicamycin (Tm, 5 µg/ml) and untreated (UT) control for 4 hr (n = 3, values are mean ± SD).

Vascular endothelial growth factor isoform A (VEGFA) is the most abundant variant of the VEGF family. Previous studies have shown that VEGFA is upregulated in human retinal ARPE-1 cells when treated with tunicamycin, an ER stress inducer [12]. To determine whether VEGFA expression is increased by other ER stress inducers in other cell types including cancer cells, we treated prostate cancer cell line, PC-3 (Figure 1A), liver cancer cell line, HepG2 (Figure 1B), and the insulinoma cells, INS-1 832/13 (Figure 1C), with two ER stress inducers, thapsigargin and tunicamycin for 4 hr. We found that VEGFA mRNA expression was increased 2–5 fold by ER stress. We confirmed that these cells were under ER stress by measuring expression levels of typical ER stress markers, spliced XBP-1 and BiP.

### HIF1α Is Not Involved in ER Stress-Mediated VEGFA Induction

**Figure 2:**
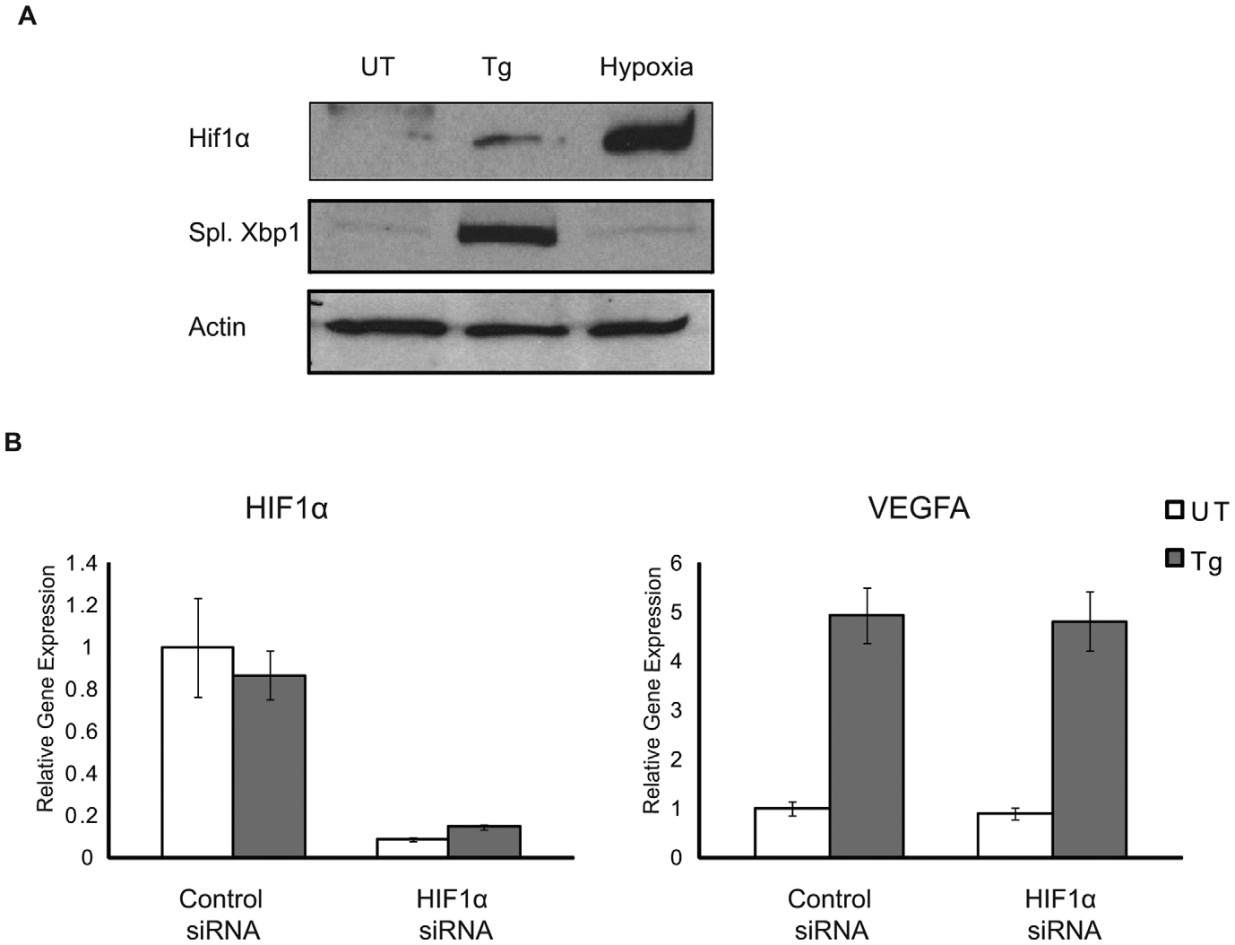
Hif1α is not required for VEGFA induction under ER stress. (**A**) HepG2 cells were treated with either thapsigargin (Tg, 1 µM) 5 hr or hypoxia (0.5% O_2_) for 24 hrs. Nuclear lysates were extracted and analyzed using anti-Hif1α and anti-Xbp1 antibodies. Spliced Xbp1 (55 kD) band is shown here. (**B**) HepG2 cells were transfected with scramble (Control) or Hif1α siRNA. 18 hrs post-transfection, cells were treated with thapsigargin (Tg, 1 µM) for 4 hrs. Total mRNA was collected and expression levels of Hif1α and VEGFA were measured by quantitative PCR (n = 3, values are mean ± SD).

HIF1α is a major regulator of VEGFA mRNA expression under hypoxia [13]. To test if HIF1α is also involved in ER stress-mediated VEGF mRNA induction, we treated HepG2 cells with either the ER stress inducer thapsigargin or hypoxia, and then measured HIF1α protein expression. HIF1α protein expression by thapsigargin treatment was much lower than that by hypoxia (Figure 2A), raising the possibility that HIF1α is not involved in ER stress-mediated VEGFA induction. To test this possibility, we knocked down HIF1α expression using siRNA directed against HIF1α in HepG2 cells, stimulated them with thapsigargin, and then measure VEGFA induction. As we predicted, siRNA-mediated knockdown of HIF1α did not affect VEGFA induction by thapsigargin (Figure 2B). These results strongly suggest that HIF1α is not a major regulator of VEGFA mRNA expression under ER stress conditions.

### IRE1α Regulates VEGFA under ER Stress Conditions via XBP-1 Splicing

**Figure 3:**
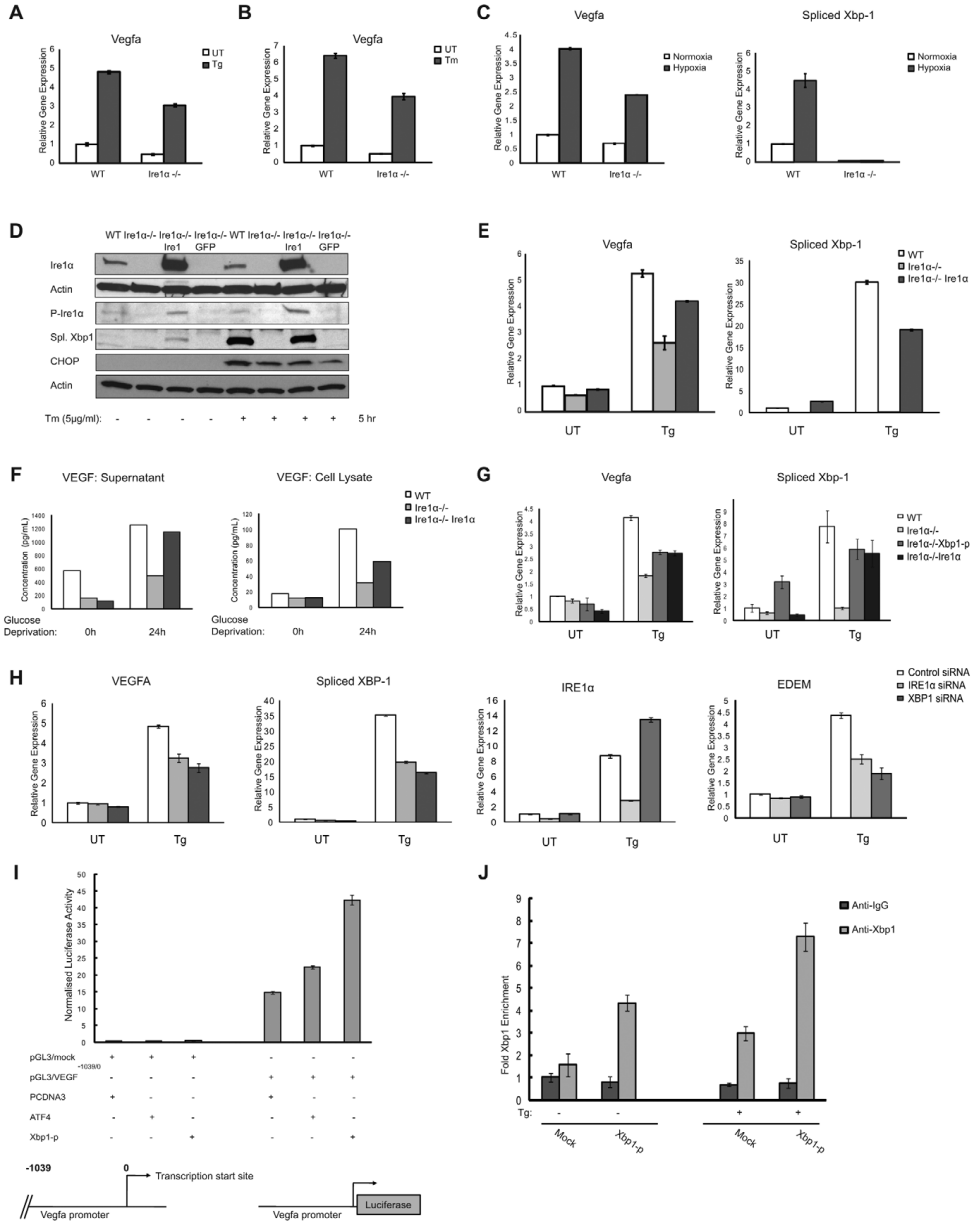
ER stress-induced VEGFA expression depends on Ire1. (**A–C**) WT and Ire1α−/− mouse embryonic fibroblasts (MEFs) were treated with ER stress inducers thapsigargin (Tg, 1 µM) 5 hr, tunicamycin (Tm, 5 µg/ml) 5 hr and hypoxia (0.5% O_2_) for 24 hr. Total mRNA was collected and VEGFA and spliced Xbp1 expression levels were measured by quantitative PCR (n = 3, values are mean ± SD). (**D**) Ire1α−/− MEFs were stably transduced with LV/Ire1α or LV/GFP, a lentivirus constitutively expressing Ire1α or GFP. These cells along with WT and Ire1α−/− MEFs were treated with thapsigargin (Tg, 1 µM) for 5 hrs. Cytoplasmic lysates were analyzed using anti-Ire1α and anti-phosphorylated activated Ire1α (P-Ire1α) antibodies. Nuclear lysates were extracted and analyzed by anti-Xbp1 to show rescue of Ire1 signaling pathway (55 kD spliced form shown here) and anti-CHOP antibodies. (**E**) Total mRNA from (D) was analyzed for VEGFA and spliced Xbp1 expression levels (n = 3, values are mean ± SD). (**F**) WT, Ire1α−/− and Ire1α−/− rescued MEFs were kept in media with or without glucose for 24 hrs. Supernatant medium as well as whole cell lysates were collected and analyzed for VEGF protein concentration by ELISA. (**G**) Ire1α−/− MEFs transfected with processed-Xbp1 along with WT, Ire1α−/− and Ire1α−/− MEFs overexpressing Ire1α protein were treated with thapsigargin (Tg, 1 µM) for 5 hrs. Total mRNA was analyzed for VEGFA and spliced Xbp1 expression levels (n = 3, values are mean ± SD). (**H**) HepG2 cells were transfected with scramble (Control), Ire1 siRNA or Xbp1 siRNA. 18 hrs post-transfection, cells were treated with thapsigargin (Tg, 1 µM) for 4 hr. Total mRNA was collected and VEGFA, spliced Xbp1, IRE1α and EDEM expression levels were measured by quantitative PCR (n = 3, values are mean ± SD). (**I**) Luciferase activity in 293T cells transfected with VEGFA-promoter reporter (pGL3/VEGF^−1039/0^) or control pGL3 mock constructs along with indicated proteins and control PCDNA3 vector (values are mean ± SD). Xbp1-p represents spliced constitutively active form of Xbp1 transcription factor. Bottom diagram shows schematic of mouse VEGFA gene as well as size of mouse VEGFA promoter construct. (**J**) Chromatin IP in Mouse neuro2a cells transfected with either mock pFlag-CMV-2 or processed-Xbp1 constructs and treated with or without thapsigargin (Tg, 1 µM) for 6 hrs was analyzed using quantitative PCR. PCR primer pair corresponded to mouse VEGFA promoter segment. Fold enrichment was quantified using ratio of amplification of PCR product relative to 10% input DNA. Value obtained from mock was defined as 1(n = 3, values are mean ± SD).

IRE1α, a key regulator of the UPR, is involved in transcriptional regulation of genes upregulated by ER stress. It has been shown that IRE1α signaling is involved in VEGFA mRNA expression by glucose deprivation and hypoxia [5]. Glucose deprivation is known to activate the UPR. We were therefore interested in determining whether IRE1α was required for VEGFA mRNA expression under ER stress conditions. VEGFA mRNA expression levels were significantly decreased in *Ire1α^−/−^* mouse embryonic fibroblasts (MEFs) as compared to those in control wild-type MEFs under various ER stress conditions induced by thapsigargin (Figure 3A), tunicamycin (Figure 3B), and hypoxia (0.5% O_2_) (Figure 3C). ER stress during hypoxia was confirmed in wild-type MEFs by measuring upregulation of spliced Xbp-1 which was absent in *Ire1α^−/−^* cells (Figure 3C, right panel). It has been shown that IRE1α is involved in the degradation of mRNAs encoding secretory proteins [14], [15] [16] [17]. To determine whether IRE1α is involved in VEGFA mRNA stability under ER stress conditions, we used actinomycin D to attenuate mRNA transcription, then challenged *Ire1α^−/−^* and control cells with thapsigargin to induce VEGFA mRNA degradation. We found that IRE1α was not involved in ER stress-mediated VEGFA mRNA decay (Figure S1). To further confirm the relationship between IRE1α signaling and VEGFA mRNA induction, we established *Ire1α^−/−^* cells transduced with lentivirus expressing human IRE1α. *Ire1α^−/−^* cells rescued with human IRE1α could activate XBP-1 splicing, indicating that ectopically expressed IRE1α was functional (Figure 3D). In *Ire1α^−/−^* cells rescued human IRE1α, VEGFA mRNA expression was restored under ER stress conditions (Figure 3E). IRE1α-mediated induction of VEGFA mRNA also enhanced VEGFA protein production as well as VEGFA secretion (Figure 3F).

IRE1α mediates XBP-1 mRNA splicing, which leads to production of an active transcription factor XBP-1-p, raising the possibility that XBP-1-p was involved in the regulation of VEGFA mRNA expression under ER stress conditions. To test this idea, we measured expression levels of VEGFA in *Ire1α^−/−^* MEFs transfected with mouse processed-XBP1 plasmid. Ectopic expression of XBP-1-p restored VEGFA induction in *Ire1α^−/−^* MEFs under ER stress conditions (Figure 3G, left panel). Although expression levels of VEGFA and spliced XBP-1 were lower in *Ire1α^−/−^* MEFs ectopically expressing XBP-1p and IRE1α as compared to control wild-type MEFs, the degree of VEGFA mRNA induction was strongly correlated with an increase in spliced Xbp1 mRNA expression (Figure 3G), suggesting that XBP-1-p-mediated induction of VEGFA is dose-dependent. To further confirm this idea, we compared expression levels of VEGFA in HepG2 cells transfected with siRNA directed against IRE1α, XBP-1, or control scramble siRNA under ER stress induced by thapsigargin. As we predicted, VEGFA mRNA induction was attenuated in the cells transfected with IRE1α siRNA and Xbp1 siRNA as compared to the cells transfected with control scramble siRNA (Figure 3H). Suppression of IRE1 and XBP1 signaling was confirmed by the downregulation of EDEM, which is a known downstream effector of XBP-1 [18].

The VEGFA promoter region contains one putative XBP-1-p binding site, the ACGT core [19], [20], 972 bp upstream of the mouse VEGFA start site, raising the possibility that XBP-1-p binds to VEGFA promoter. To test this idea, we derived a reporter construct by cloning 1 kb upstream sequences of the mouse VEGFA transcription start site upstream of the luciferase gene. Ectopic XBP-1-p expression increased activity of VEGFA promoter-luciferase (Figure 3I). In order to determine direct interaction of XBP1 with VEGFA, we did a chromatin immunoprecipitation (ChIP) analysis of the mouse VEGFA promoter. Mouse neuro2A cells either mock transfected or transfected with XBP-1-p, were treated with thapsigargin. ChIP analysis indicates that XBP-1-p was recruited to the mouse VEGFA promoter under ER stress conditions in vivo (Figure 3J). XBP-1-p overexpression improves binding efficiency which is further increased by ER stress thus supporting the idea of dose-dependent upregulation of VEGFA transcription under ER stress. Collectively, the results of Figure 3 show that VEGFA is a direct target of the IRE1α-XBP-1 pathway.

### IRE1α Regulates VEGFA Expression in Labyrinthine Trophoblast Cells

**Figure 4:**
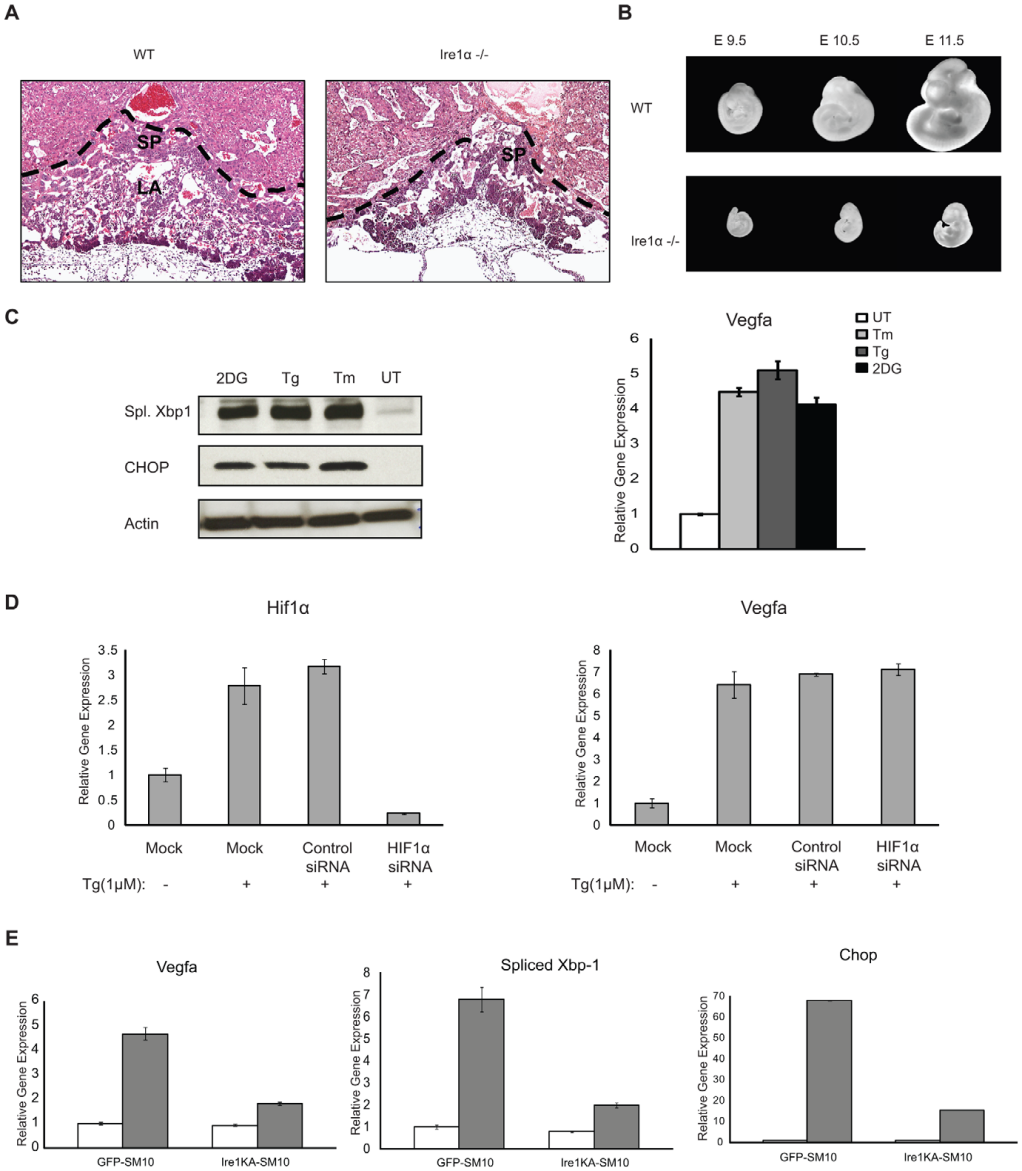
Ire1α−/− mouse labyrinthine placenta defect is due to Ire1-dependent VEGFA expression. (**A**) WT and Ire1α−/− placenta were collected at d10.5 and vertically dissected and HE stained to show all placenta layers from maternal decidua to fetal chorionic plate SP =  Spongiotrophoblast, LA =  Labyrinthine trophoblast. (**B**) WT and Ire1α−/− embryo littermates were collected from Ire1α+/− females mated with Ire1α+/− males. Ire1α−/− animals die by embryonic day 10.5. (**C**) Labyrinthine trophoblast SM10 cells were treated with thapsigargin (Tg, 1 µM) tunicamycin (Tm, 5 µg/ml) or 2-Deoxy-glucose (2 DG) for 6 hrs. Total cell lysates and mRNA were collected. Lysates were analyzed by anti-Xbp1 showing the spliced active 55KD band, and anti-CHOP antibody (left panel). Total mRNA was used for expression analysis of VEGFA (right panel) (n = 2, values are mean ± SD). (**D**) SM10 cells were transfected with scramble (Control) or Hif1α siRNA. 18 hrs post-transfection, cells were treated with thapsigargin (Tg, 1 µM) for 4 hrs. Total mRNA was collected and expression levels of HIF1α and VEGFA were measured by quantitative PCR (n = 3, values are mean ± SD). (**E**) SM10 cells were stably transduced with either LV/control GFP or LV/Ire1KA mutant lentivirus to constitutively express GFP or Ire1KA protein. Transduced stably expressing cells were kept in media with or without glucose for 24 hrs. Total mRNA was then analyzed for VEGFA, spliced XBP1 and CHOP expression (n = 2, values are mean ± SD).

Our group and others have previously reported that *Ire1α^−/−^* mice are embryonic lethal by embryonic day 12.5 [21], [22], [23]. It has been reported that the embryonic lethality of *Ire1α^−/−^* mice is due to the defect in the development of the labyrinthine trophoblast cells which are critical for integration and exchange between fetal and maternal blood vessels in the placenta [23]. It has previously been shown that loss of a single *Vegfa* allele in mice can result in embryonic lethality by embryonic day 11.5 due to a defect in the development of labyrinthine trophoblast cells [24]. We therefore considered the possibility that IRE1α has a role in the survival of labyrinthine trophoblast cells through the activation of VEGFA expression. As reported previously, we found that *Ire1α^−/−^* placentas failed to develop proper labyrinthine trophoblast on embryonic day 10.5 (Figure 4A). Because of the progressive loss of blood vessels in the labyrinthine layer, *Ire1α^−/−^*embryos were comparatively smaller in size than wild-type embryos and did not survive more than embryonic day 12.5 (Figure 4B and Table 1). To confirm the role of IRE1α signaling in mouse labyrinthine trophoblast cells, we also analyzed the mouse labyrinthine trophoblast cell line, SM10 [25]. These cells responded robustly to ER stress inducers, tunicamycin and thapsigargin as well as 2-deoxy glucose which causes glucose deprivation, as indicated by upregulation of spliced Xbp1 and Chop protein expression levels (Figure 4C, left panel). VEGFA mRNA was also induced significantly upon ER stress (Figure 4C, right panel). During embryonic development, HIF1α is activated in the growing placenta [8]. To confirm that HIF1α was not involved in ER stress-mediated VEGFA mRNA induction in SM10 cells, we knocked down HIF1α expression by siRNA and measured VEGFA mRNA expression. As expected, RNAi-mediated knockdown of HIF1α did not affect VEGFA mRNA induction by thapsigargin treatment in SM10 cells (Figure 4D).

**Table 1:** Mouse embryo survival data.

Age (days)	Total No.	*Ire1α +/+*	*Ire1α +/−*	*Ire1α −/−*	Resorbed
**E 8.5**	35	4 (11%)	21 (60%)	8 (23%)	2
**E 9.5**	34	8 (24%)	19 (56%)	4 (12%)	3
**E10.5**	84	19 (23%)	45 (54%)	15 (18%)	5
**E11.5**	34	8 (24%)	17 (50%)	6 (18%)	3
**E12.5**	33	7 (21%)	14 (42%)	2 (6%)^a^	10
**E13.5**	25	6 (24%)	14 (56%)	2 (8%)	3

Ire1+/− heterozygous animals were crossed and embryos as well as placenta were collected at various stages of development starting at embryonic day 8.5.

We next sought to verify that ER stress-mediated VEGFA mRNA induction required IRE1α in SM10 cells. We transduced SM10 cells with lentivirus expressing a kinase inactive K599A mutant form of IRE1α, which has been reported to act as a dominant-negative of wild-type IRE1α [16], [26], [27], or GFP as a control, then we induced physiological ER stress using glucose deprivation which normally occurs during placenta development [6], [7], [8]. In cells expressing kinase inactive K599A mutant form of IRE1, VEGFA mRNA levels were decreased as compared to control cells expressing GFP (Figure 4E). The suppression of IRE1α signaling by the kinase inactive IRE1α K599A mutant was confirmed by the attenuation of XBP-1 splicing (Figure 4E). Collectively, these results indicate that Ire1 has an essential function in VEGFA expression in the labyrinthine trophoblast cells which is independent of HIF1α activity.

### ER Stress-Induced VEGFA Upregulation Is Also Dependent on PERK

As described above, IRE1α-XBP-1 signaling plays a role in the induction of VEGFA mRNA under ER stress conditions. However, the lack of IRE1α only partially attenuated VEGFA mRNA induction. Therefore, it is possible that other components of the UPR also play a role in VEGFA mRNA expression. To test this idea, we next sought to study the role of PERK signaling in VEGFA mRNA expression.

**Figure 5:**
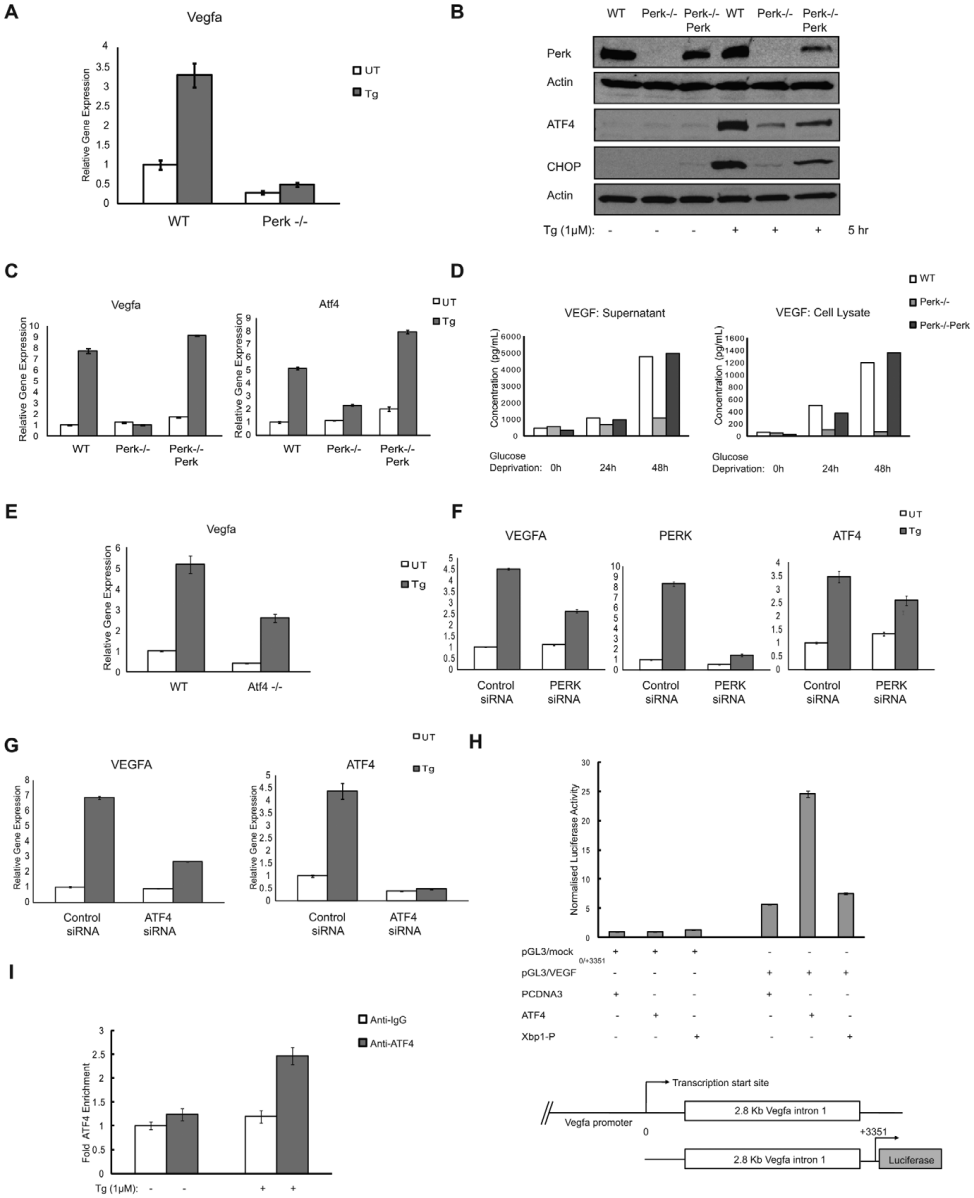
ER stress induced VEGFA upregulation is also dependent on Perk. (**A**) WT and Perk−/− MEFs were treated with thapsigargin (Tg, 1 µM) for 5 hrs. Total mRNA was collected and VEGFA expression levels were measured by quantitative PCR (n = 3, values are mean ± SD). (**B**) Perk−/− MEFs were stably transduced with LV/Perk, a lentivirus constitutively expressing Perk. These cells along with WT and Perk−/− MEFs and treated with thapsigargin (Tg, 1 µM) for 5 hrs. Cytoplasmic protein lysates were extracted and analyzed by immunoblot using anti-Perk antibody. Nuclear protein lysates were analyzed by immunoblot using anti-ATF4 and anti-Chop antibodies to show rescue of Perk signaling pathway. (**C**)Total mRNA from (B) was analysed for VEGFA and ATF4 expression levels (n = 3, values are mean ± SD). (**D**) WT, Perk−/− and Perk−/− rescued MEFs were kept in media with or without glucose for 0, 24 and 48 hrs. Supernatant medium as well as whole cell lysates were collected and analyzed for VEGF protein concentration by ELISA. (**E**) ATF4−/− MEFs were treated with thapsigargin (Tg, 1 µM) for 4 hrs. Total mRNA was collected and VEGFA expression levels were measured by quantitative PCR (n = 3, values are mean ± SD). (**F**) HepG2 cells were transfected with scramble (Control) and Perk siRNA. 18 hrs post-transfection, cells were treated with thapsigargin (Tg, 1 µM) for 4 hr. Total mRNA was collected and VEGFA, PERK and ATF4 expression levels were measured by quantitative PCR (n = 3, values are mean ± SD). (**G**) HepG2 cells were transfected with scramble (Control) and ATF4 siRNA and treated with thapsigargin (Tg, 1 µM) for 4 hr. Total mRNA was collected and VEGFA and ATF4 expression levels were measured by quantitative PCR (n = 3, values are mean ± SD). (**H**) Luciferase activity in 293T cells transfected with mouse VEGFA-intron 1 reporter (pGL3/VEGF^0/+3351^) or control pGL3 mock constructs along with indicated proteins and control PCDNA3 vector (values are mean ± SD). Bottom diagram shows schematic of mouse VEGFA gene as well as size of mouse VEGFA- intron construct. (**I**) Chromatin IP in Mouse neuro2a cells treated with or without thapsigargin (Tg, 1 µM) for 6 hrs was analyzed using quantitative PCR. PCR primers corresponded to mouse VEGFA-intron 1 genomic region. Fold enrichment was quantified using ratio of amplification of PCR product relative to 10% input DNA. Value obtained from mock was defined as 1(n = 3, values are mean ± SD).

In *Perk^−/−^* MEFs, VEGFA mRNA induction by thapsigargin treatment was significantly attenuated as compared to wild-type MEFs (Figure 5A). To confirm that the decreased expression of VEGFA was due to lack of Perk expression, we analyzed the effect of Perk-rescue in *Perk^−/−^* cells. We transduced *Perk^−/−^* MEFs with lentivirus expressing PERK. To confirm that ectopically expressed PERK was functional, we treated the rescued cells along with wild-type and *Perk^−/−^* parental cells with thapsigargin and measured ATF4 and CHOP protein expression, two proteins regulated by PERK. As expected, ATF4 and CHOP protein expression levels were restored in the rescued cells (Figure 5B). VEGFA mRNA expression levels were also restored to wild-type levels under ER stress conditions in the rescued cells (Figure 5C). VEGF protein expression as well as secretion were also restored in the rescued cells (Figure 5D).

Previous studies have shown ATF4 translation is dependent on PERK and suggested that ATF4 plays a role in VEGF mRNA expression under oxidative stress [28], [29]. These considerations prompted us to investigate the role of ATF4 in VEGFA mRNA expression under ER stress conditions. We measured VEGFA mRNA expression in *Atf4^−/−^* MEFs and wild-type MEFs. Figure 5E shows that VEGFA mRNA levels were attenuated in *Atf4^−/−^* cells as compared to wild-type cells.

To further confirm the role of the PERK-ATF4 pathway in VEGFA mRNA induction, we transfected HepG2 cells with siRNAs directed against PERK and ATF4 and then measure VEGFA mRNA expression under ER stress conditions. RNAi-mediated knockdown of PERK and ATF4 in HepG2 cells resulted in a significant decrease of VEGFA mRNA expression (Figure 5F and G).

ATF4 has been previously shown to interact with first intron of the human VEGFA gene under oxidative stress conditions caused by arsenite treatment [28]. ATF4 interacts with an amino-acid response element (AARE) present in the intron. We therefore considered the possibility that ATF4 binds to the first intron of VEGFA gene under ER stress conditions.

To test this idea, a reporter plasmid carrying a 3.3 Kb fragment of intron 1 of the mouse VEGFA gene was cloned into pGL3 luciferase vector. Ectopic ATF4 expression, but not XBP-1, increased the activity of the reporter (Figure 5H). ChIP analysis was performed on neuro2A cells in order to confirm the interaction. The ChIP PCR product comprised of 109 bp starting from +1581 of the 2891 bp mouse VEGFA intron1.The ChIP experiments revealed that following addition of thapsigargin, ATF4 was recruited to the VEGFA intron 1 (Figure 5I). Collectively, these results indicate that the PERK-ATF4 pathway directly regulates VEGFA mRNA expression through intron 1 of VEGFA gene under ER stress condtions.

### ATF6α Also Plays a Role in VEGFA mRNA Upregulation under ER Stress

**Figure 6:**
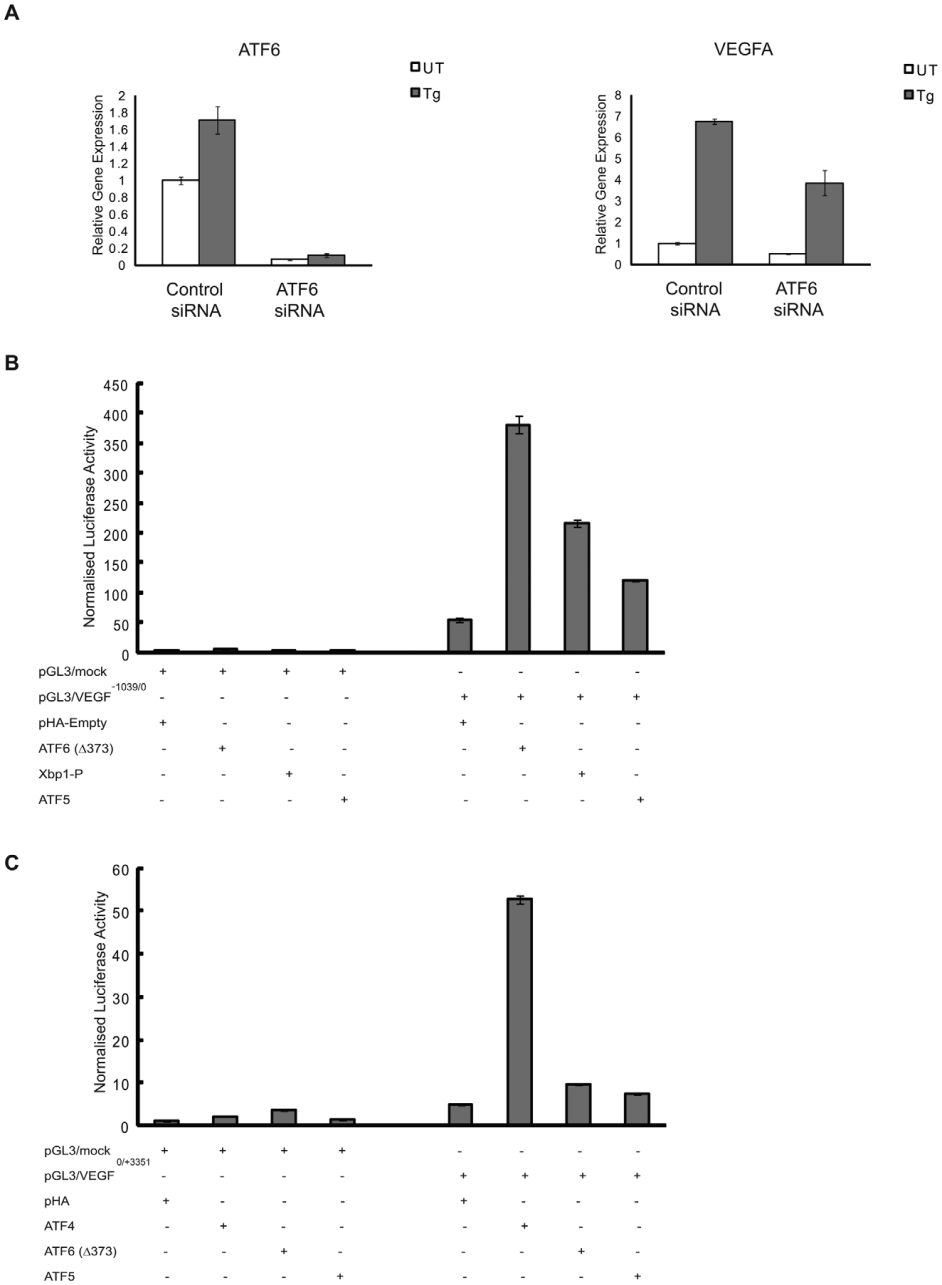
ATF6 has an active role in ER stress-induced VEGFA upregulation. (**A**) HepG2 cells were transfected with scramble (Control) and ATF6 siRNA and treated with thapsigargin (Tg, 1 µM) for 4 hr. Total mRNA was collected and VEGFA and ATF6 expression levels were measured by quantitative PCR (n = 3, values are mean ± SD). (**B**) Luciferase activity in 293T cells transfected with VEGFA-promoter reporter (pGL3/VEGF^−1039/0^) or control pGL3 mock constructs along with indicated proteins and control pHA vector (values are mean ± SD). ATF6(Δ373) represents cleaved constitutive active form of ATF6 transcription factor. Xbp1-p and ATF5 represent positive and negative controls respectively. (**C**) Luciferase activity in 293T cells transfected with mouse VEGFA-intron 1 reporter (pGL3/VEGF^0/+3351^) or control pGL3 mock constructs along with indicated proteins and control pHA vector (values are mean ± SD). ATF4 and ATF5 serve as positive and negative controls respectively.

ATF6α, an ER transmembrane protein, is another upstream component of the UPR. Under ER stress conditions ATF6α translocates from ER to the Golgi where its cytoplasmic domain is cleaved from the membrane to produce a potent transcription factor regulating UPR target genes [30]. XBP-1 is one of the targets of cleaved-ATF6α [31]. Hence, we tested the possibility if ATF6α was involved in VEGFA mRNA expression under ER stress conditions. As predicted, RNAi-mediated knockdown of ATF6α in HepG2 cells resulted in a significant decrease of VEGFA mRNA expression under ER stress conditions (Figure 6A). To further establish a relationship between ATF6α and VEGFA mRNA induction, we tested if ATF6α could increase the activity of gene regulatory regions of VEGFA gene. Ectopic expression of cleaved active ATF6α increased the activity of VEGFA-promoter reporter (Figure 6B), but not VEGFA-intron reporter (Figure 6C). Collectively, these results strongly suggest that ATF6α also plays role in VEGFA mRNA induction by binding its promoter under ER stress conditions.

## Discussion

**Figure 7:**
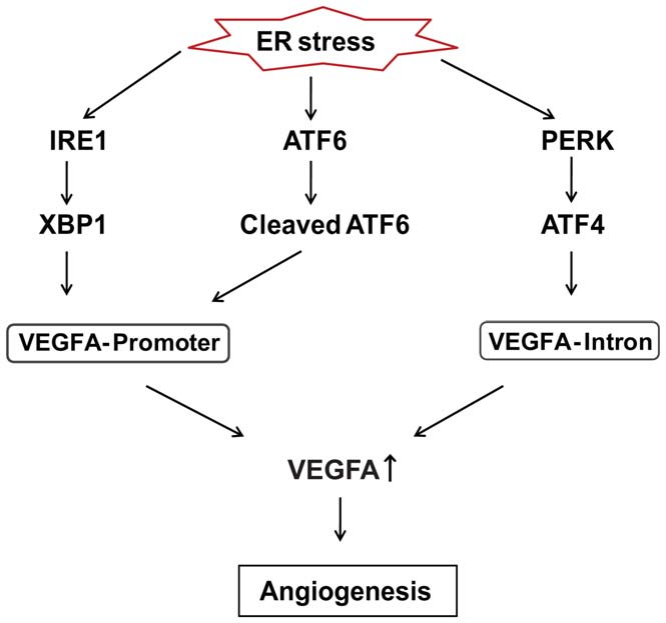
VEGFA expression is regulated by ER stress. ER stress signaling regulates VEGFA expression via its principal regulators Ire1α, Perk and ATF6α. Proposed pathway shows how ER stress signaling might affect growth and survival of both developing placenta as well as tumor through angiogenesis. This is accomplished by activating VEGFA transcription via different regulatory regions on the VEGFA gene.

Our results indicate that the UPR is activated not only by ER stress but also deprivation of nutrients and oxygen, and robustly regulates VEGFA mRNA expression. The regulation of VEGFA by ER stress is mediated by UPR master regulators, IRE1α, PERK and ATF6α via their respective transcription factors, XBP-1, ATF4 and cleaved-ATF6 respectively (Figure 7). Our results show that VEGFA induction by ER stress is independent of the hypoxia inducible factor 1 (HIF-1) pathway, a major signaling pathway for VEGFA induction by hypoxia [13] or there is a cross-talk between the XBP-1, ATF4, ATF6α, and HIF-1 pathways. The robust induction of VEGFA by IRE1α signaling is also important for the development and survival of labyrinthine trophoblast cells in the placenta.

VEGFA is the major vascular endothelial growth factor and plays prominent roles in angiogenesis [32]. Angiogenesis is crucial to many physiological and pathological processes including development of labyrinthine trophoblast cells in the placenta [24], [33], [34]. Mammalian placenta is a vital secretory organ characterized by secretion of large amounts of diverse proteins like placental lactogens, proliferins and other growth factors required for the growing embryo. In mice, the placenta is formed by fusion of the embryo-derived allantois (i.e., umbilical cord) and the extra-embryonic chorion which derives from the polar trophectoderm overlying the embryo. Chorioallantoic attachment at embryonic day 8.5 causes chorionic trophoblast to differentiate into various layers of labyrinthine trophoblast cells. These labyrinthine trophoblast cells undergo extensive villus branching to form the labyrinth by embryonic day 10.5 [34]. The formation of this layer is critical for integration and exchange between fetal and maternal blood vessels [6], [33]. It has been reported that hypoxia and nutrient deprivation, which have been shown to activate both IRE1α and PERK in this study, are involved in various stages of placental development, one of them being trophoblast differentiation [6], [7], [8]. Our results suggest that the generation of a fully functional labyrinthine trophoblast requires a coordinated action of downstream effectors regulated by IRE1α including VEGFA which is independent of HIF1α signaling.

Angiogenesis is crucial for sustaining tumors. IRE1α signaling is known to be essential for angiogenesis and growth of tumor cells through VEGFA induction [3], [5]. PERK signaling also has been shown to be important for survival of tumor cells [35]. Our data show that the IRE1α-XBP-1, PERK-ATF4, and ATF6α pathways can independently regulate VEGFA mRNA expression under ER stress conditions via different regulatory regions of the VEGFA gene. Our results show that both VEGFA promoter as well as the intron is engaged simultaneously by UPR effectors under ER stress conditions. This tells us that coordinated activation of all three regulators is essential for proper upregulation of VEGFA mRNA expression. Inactivation or knockdown of any of these three master regulators impairs VEGFA upregulation under ER stress.

Many downstream target genes of the UPR are co-regulated by IRE1α, PERK, and ATF6α, ensuring the redundancy and robustness of this adaptive response. However, this biological design that mediates robustness also has drawbacks. In cancer cells, VEGFA induction mediated by the UPR is used to maintain abnormal ER homeostasis and help these cells to survive and proliferate under chronic ER stress conditions caused by nutrient deprivation and hypoxia. Thus, inhibiting IRE1α, PERK, and ATF6α is a promising novel therapeutic modality for cancer treatment.

## Materials and Methods

### Cell Culture

Hepatocellular cell line, HepG2, and human prostate cancer cell line, PC3, were obtained from ATCC. HepG2 was maintained in MEM with supplements according to ATCC cell culture instructions. PC3 was maintained in DMEM with 10% FBS. Rat insulinoma cells, INS-1 832/13, were a gift from Dr. Christopher Newgard (Duke University Medical Center) and cultured in RPMI 1640 supplemented with 10% FBS. *Ire1α^−/−^* and *Perk^−/−^* mouse embryonic fibroblasts were gifts from Dr. David Ron (New York University School of Medicine) and maintained in DMEM 10% FBS. Mouse labyrinthine trophoblast SM10 cells were a gift from Dr. Joan Hunt (University of Kansas) and maintained in RPMI 1640 supplemented with 2 mM glutamine, 1% sodium pyruvate, 5×10^−5^ M 2-Mercaptoethanol and 10% FBS. Human embryonic kidney 293T cells and Mouse neuro2a cells were obtained from ATCC and maintained in DMEM with 10% FBS.

### Plasmids

Mouse Atf4, mouse Xbp1-processed, mouse Perk, human Ire1α and human Ire1αKA (K599A) dominant-negative kinase mutant plasmids were provided by Dr. David Ron (New York University School of Medicine). Mouse ATF5 plasmid was a gift from Dr. Michael Green (University of Massachusetts Medical School). Cleaved processed ATF6(▵373) plasmid was provided by Dr. R. Prywes (Columbia University).

### Lentivirus System

Lentivirus expressing human IRE1α, human IRE1αKA and mouse Perk and GFP were generated by subcloning these fragments into lentiviral expression plasmid, pLenti-CMV/TO (Dr. Eric Campeau at the University of Massachusetts Medical School). Lentivirus was produced in HEK293T cells by transfection using Lipofectamine2000 (Invitrogen Carlsbad, CA). Lentiviral supernatant was collected 48 hours after transfection, and stored at −80°C. Details of this lentivirus system were described previously [36]. *Ire1α^−/−^* and *Perk^−/−^* cells were infected with human IRE1α and mouse Perk lentivirus respectively and selected with puromycin 2 µg/ml to generate stable cell lines. SM10 cells were infected with human IRE1αKA and control GFP lentivirus and selected with Puromycin 1 µg/ml to generate stable cell lines.

### siRNA and Plasmid Transfection

Small interfering RNA (siRNA) was transfected using the Nucleofector Device (Amaxa Biosystems, Gaithersburg, MD) into HepG2 cells according to manufacturer recommendations. Human HIF1-α smart pool siRNA was obtained from Dharmacon, (Lafayette, CO). SiRNAs directed against human PERK, human IRE1α, human ATF4 and human XBP1 were synthesized by IDT (Coralville, IA): for human PERK: CCAGAGAAGTGGCAAGAAA; for human IRE1: AGACAGAGGCCAAGAGCAA; for human ATF4: GCAAAGAGCTGGAAAAGAA; for human XBP1: GGTATTGACTCTTCAGATT. Cells were incubated in media for overnight after siRNA transfection, and then put under ER stress conditions. Mouse XBP1-processed plasmid was transfected using the Nucleofector device (Amaxa Biosystems, Gaitherburg, MD) into mouse embryonic fibroblasts according to manufacturer recommendations.

### Western Blotting

Cells were lysed in M-PER (Pierce, Rockford IL) for whole cell protein extraction, or NE-PER (Pierce, Rockford IL) for nuclear and cytoplasmic protein extraction, after adding protease inhibitor (Sigma, Saint Louis, MO) according to supplier protocol. Lysates were run on a 4%–20% linear gradient SDS-PAGE (BioRad, Hercules, CA) gel. Anti-Atf4 (anti-CREB-2 H-290), Anti-Xbp1, anti- CHOP/GADD153 and anti-Actin antibodies were obtained from Santa Cruz Biotechnology (Santa Cruz, CA). Anti-Ire1α, anti-Perk antibodies was obtained from Cell Signaling (Danvers, MA). Anti-Hif1α, Anti-phospho Ire1 antibodies was obtained from Novus Biologicals (Littleton, CO).

### Quantitative Polymerase Chain Reaction

Total RNA was isolated from the cells by using the RNeasy Mini Kit (Qiagen, Valencia, CA). 1 µg of total RNA from cells was reverse transcribed with Oligo-dT primer (Promega, Madison, WI). For the thermal cycle reaction, the iQ5 system (BioRad, Hercules, CA) was used at 95°C for 10 min, then 40 cycles at 95°C for 10 sec, and at 55°C for 30 sec. The relative amount for each transcript was calculated by a standard curve of cycle thresholds for serial dilutions of cDNA sample and normalized to the amount of the house-keeping beta-actin levels. The polymerase chain reaction (PCR) was performed in triplicate for each sample; all experiments were repeated three times. Power SYBR Green PCR Master Mix (Applied Biosystems, Foster City, CA) was used for the quantitative PCR. PCR primer sequences are listed in Table S1.

### mRNA Stability Assay

Cellular mRNA transcription was attenuated by treating cells with 5 µg/ml Actinomycin D (Sigma A-4262) for 1 hr. followed by treatment with thapsigargin (0.2 µM) for different times. Total RNA was collected and quantitative PCR method described above was employed to measure levels of VEGFA gene transcripts. Time point zero for each condition was standardized to 1 and the subsequent rate of degradation of mRNA was measured.

### Luciferase Assay

Mouse Vegfa 1 Kb (−1039 to 0) promoter and 3.3 Kb Intron (460 bp Exon 1+2891 bp Intron 1) was cloned from Mouse BAC clone RP23 (Invitrogen, Carlsbad, CA) into Xho1/HindIII site of pGL3 luciferase vector (Promega, Madison, WI). 293T cells were transfected with both reporters as well as mock pGL3 vector along with Atf4, processed-Xbp1, processed-Atf6 (Δ373) and Atf5 by Lipofectamine™ 2000 (Invitrogen, Carlsbad, CA). Twenty-four hrs post-transfection, lysates were prepared using a Luciferase Assay System kit (Promega, Madison, WI) according to manufacturer's protocol. The light produced from the samples was read by a standard plate reading luminometer. Each sample was read in triplicate and normalized against the signal produced from mock wells. β-galactosidase activity was measured by β-Gal Reporter Gene Assay, chemiluminescent (Roche Diagnostics, Mannheim, Germany). The assay was performed independently three times.

### Chromatin Immunoprecipitation (ChIP)

Mouse neuro2a cells were transfected with pFlag-CMV-2 and processed-Xbp1 expression plasmids by Lipofectamine™ 2000 (Invitrogen, Carlsbad, CA). After 36 hrs, cells were treated with or without thapsigargin for further 6 hrs and fixed in 1% formaldehyde. Untransfected Neuro2a cells were also treated with or without thapsigargin for 6 hrs and fixed in 1% formaldehyde ChIPs were performed using SimpleChIP™ Enzymatic Chromatin IP Kit (Agarose Beads) (Cell Signaling, Danvers, MA) as per manufacturer's recommendation. Antibodies against Xbp1 and ATF4 from Santa Cruz Biotechnology (Santa Cruz, CA) as well as a negative control, rabbit IgG, were used for ChIP assay. Purified DNA from cross-linked cells was dissolved in 25 µl TE; 2 µl was used for PCR. Inputs consisted of 10% chromatin before immunoprecipitation. Quantitative PCRs were performed as described in *Quantitative polymerase chain reaction* section using following primer sets: for mouse Vegfa promoter, ATTTCCTGGGAAAGGGAATTG and TCCACGGCCTCAAAATTATC; for mouse Vegfa intron 1, GCCACAGTGTGACCTTCAGA and CGTGGAGAAAGGGAACAGAA.

### ELISA

WT, *Ire1α^−/−^*, *Perk^−/−^*, Ire1 rescued and Perk rescued mouse embryonic fibroblasts were treated with media without glucose as indicated. Both supernatant medium as well as whole cell lysates were collected. Mouse VEGF secreted protein from supernatant and lysates were measured via ELISA analysis using Bio-Plex 200 System (BioRad, Hercules, CA) at the UMass Mouse Phenotyping Center at University of Massahusetts Medical School, Worcester, MA.

### *Ire1α^+/−^* Animals

*Ire1α^+/−^* mice on 129 background are maintained at the animal medicine core at UMass Medical School (Worcester, MA).

## Supporting Information

Figure S1 Ire1α does not affect VEGF mRNA stability. WT and Ire1α−/− MEFs were treated with or without actinomycin D (5 µg/ml) for 1 hr. Cells were then treated with ER stress inducer, thapsigargin (Tg, 1 µM) for 0, 1.5 or 3 hrs. Total mRNA was collected and expression levels of VEGFA were measured by quantitative PCR (n = 3, values are mean ± SD).
(2.72 MB TIF)

Table S1 Quantitative PCR Primer Sequences. The following table indicates sets of primers used for quantitative PCR.
(0.04 MB DOC)
