# Supplementary figures and images for: Transcriptional Regulation of VEGF-A by the Unfolded Protein Response Pathway
Source: PLoS One. 2010 Mar 8;5(3):e9575. doi: 10.1371/journal.pone.0009575 (PMC2833197; doi:10.1371/journal.pone.0009575)

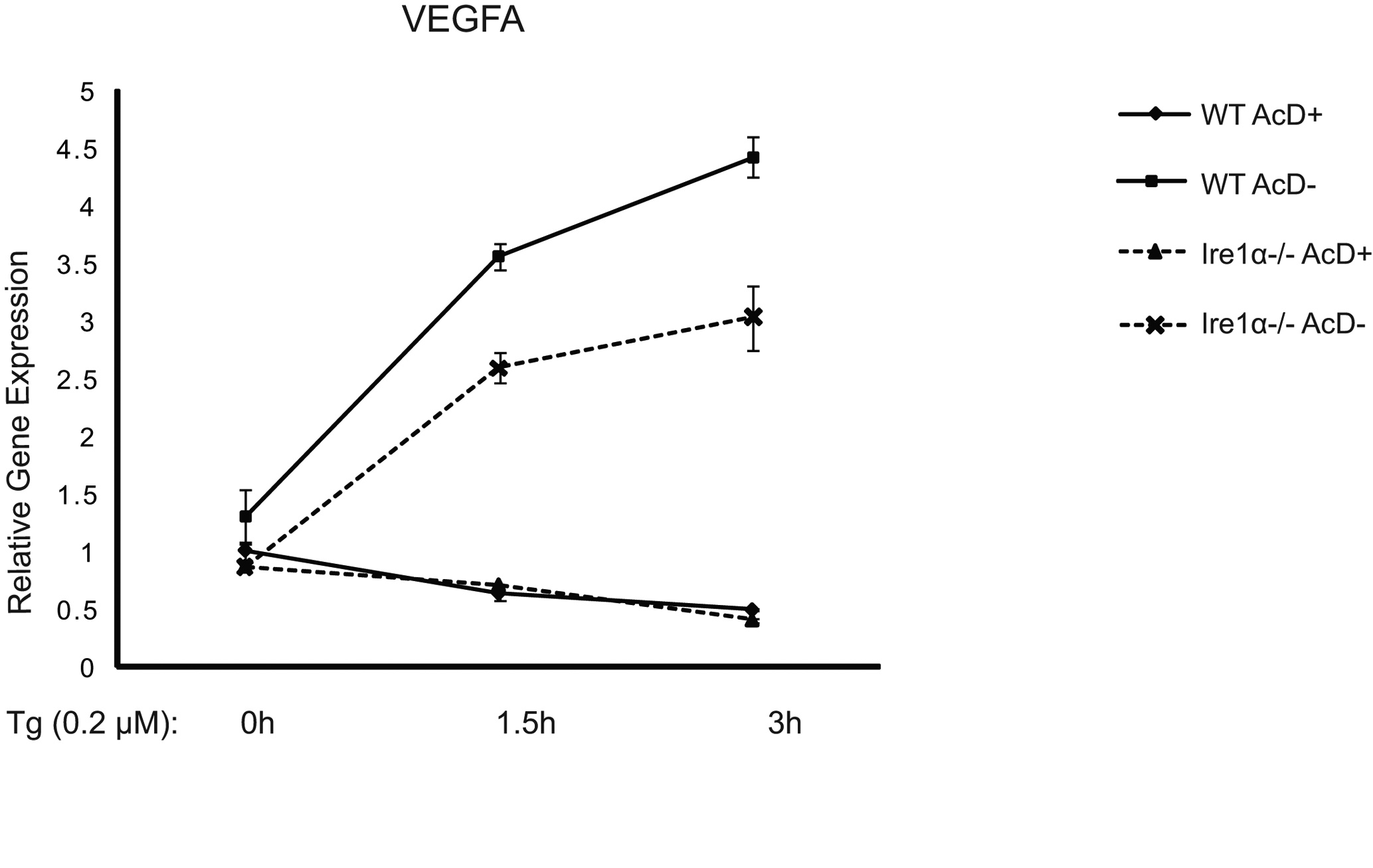

Supplement: Figure S1 — Ire1α does not affect VEGF mRNA stability. WT and Ire1α−/− MEFs were treated with or without actinomycin D (5 µg/ml) for 1 hr. Cells were then treated with ER stress inducer, thapsigargin (Tg, 1 µM) for 0, 1.5 or 3 hrs. Total mRNA was collected and expression levels of VEGFA were measured by quantitative PCR (n = 3, values are mean ± SD). (2.72 MB TIF) [file pone.0009575.s001.tif]
