# Supplementary material for: Transcriptional Regulation of VEGF-A by the Unfolded Protein Response Pathway
Source: PLoS One. 2010 Mar 8;5(3):e9575. doi: 10.1371/journal.pone.0009575 (PMC2833197; doi:10.1371/journal.pone.0009575)
Supplement: Table S1 — Quantitative PCR Primer Sequences. The following table indicates sets of primers used for quantitative PCR. (0.04 MB DOC) [file pone.0009575.s002.doc]

**Table S1. Quantitative PCR Primer Sequences.**

| **Species** | **Gene** | **Forward Sequence 5'-3'** | **Reverse Sequence 5'-3'** |
| --- | --- | --- | --- |
| Mouse | Actin | GCAAGTGCTTCTAGGCGGAC | AAGAAAGGGTGTAAAACGCAGC |
| Mouse | Vegfa | TGAAGCCCTGGAGTGCGT | AGGTTTGATCCGCATGATCTG |
| Mouse | Atf4 | GGGTTCTGTCTTCTCCA | AAGCAGCAGAGTCAGGCTTC |
| Mouse | Spl. Xbp1 | CTGAGTCCGAATCAGGTGCAG | GTC CATGGGAAGATGTTCTGG |
| Mouse | Chop | CCACCACACCTGAAAGCAGAA | AGGTGAAAGGCAGGGACTCA |
| Mouse | Hif1α | GATTCGCCATGGAGGGC | TTCGACGTTCAGAACTCATCTTTT |
| Rat | Actin | GCAAATGCTTCTAGGCGGAC | AAGAAAGGGTGTAAAACGCAGC |
| Rat | BiP | TGGGTACATTTGATCTGACTGGA | CTCAAAGGTGACTTCAATCTGGG |
| Rat | Vegfa | CAAGCCGTCCTGTGTGCC | TCCAGGGCTTCATCATTGC |
| Human | GAPDH | TGTTCGACAGTCAGCCGC | GGTGTCTGAGCGATGTGGC |
| Human | HIF1α | AGGAGGATCACCCTCTTCGT | TCTCCTCAGGTGGCTTGTC |
| Human | VEGFA | CCTTGCTGCTCTACCTCCAC | ATGATTCTGCCCTCCTCCTT |
| Human | IRE1α | GGCCTGGTCACCACAATTAGA | TTTGGGAAGCCTGGTCTCC |
| Human | PERK | CCTCACCATTTGCCTAAGGA | GGGGGACTTTCCTTCTTCTG |
| Human | ATF4 | GTTCTCCAGCGACAAGGCTA | ATCCTCCTTGCTGTTGTTGG |
| Human | Spl. XBP1 | CTGAGTCCGAATCAGGTGCAG | ATCCATGGGGAGATGTTCTGG |
| Human | BiP | TGTTCAACCAATTATCAGCAAACTC | TTCTGCTGTATCCTCTTCACCAGT |
| Human | EDEM | CAAGTGTGGGTACGCCACG | AAAGAAGCTCTCCATCCGGTC |
